# Changes in Practitioners’ Attitudes toward the Recommendations for Clinical Questions of the Japanese Guideline for Management of Hyperuricemia and Gout, Third Edition between 2018 and 2020 in the Questionnaire’s Surveys: Involving Their Attitude toward the New Questionnaires in 2020

**DOI:** 10.31662/jmaj.2024-0399

**Published:** 2025-03-21

**Authors:** Naoyoshi Otani, Toshihiro Hamada, Masanari Kuwabara, Satoshi Miyazaki, Yasuto Sato, Takeo Nakayama, Haruaki Ninomiya, Tsuneo Konta, Ichiro Hisatome

**Affiliations:** 1 Department of Cardiology, National Hospital Organization, Yonago Medical Center, Yonago, Japan; 2 Department of Cardiology, Dokkyo Medical University Nikko Medical Center, Nikko, Japan; 3 Department of Internal Medicine, Nichinan Hospital, Nichinan, Japan; 4 Division of Public Health, Center for Community Medicine, Jichi Medical University, Shimotsuke, Japan; 5 Division of Cardiovascular Medicine, Department of Medicine, Jichi Medical University, Shimotsuke, Japan; 6 Division of Cardiology, Fujii Masao Memorial Hospital, Kurayoshi, Japan; 7 Graduate School of Public Health, Shizuoka Graduate University of Public Health, Shizuoka, Japan; 8 Department of Health Informatics, Graduate School of Medicine & School of Public Health, Kyoto University, Kyoto, Japan; 9 Department of Biological Regulation, Tottori University Faculty of Medicine, Yonago, Japan; 10 Department of Public Health and Hygiene, Yamagata University School of Medicine, Yamagata, Japan

**Keywords:** clinical guideline, hyperuricemia, gout, clinical questions, urate-lowering agents

## Abstract

**Introduction:**

The Japanese Society of Gout, Uric, and Nucleic Acid developed the Japanese Guideline for Management of Hyperuricemia and Gout (JGMHG) third edition, which contains seven clinical questions (CQs) and corresponding recommendations. Questionnaire surveys were conducted to clarify how recommendations regarding CQs influence decision-making in clinical practice.

**Methods:**

The surveillances were conducted twice in 2018, just after the publication of JGMHG, and in 2020, 2 years later. The participants were members of the Japanese Society of Gout, Uric, and Nucleic Acid. While the 2018 surveys contained questionnaires on the recommendations of seven CQs as well as three questionnaires on the status of their use, the 2020 surveys contained those regarding their recommendations of seven CQs and seven questionnaires on the status of their use. The answers from 74 (response rate: 16%) and 61 (response rate: 14%) participants in 2018 and 2020, respectively, were analyzed using the chi-square test or Fisher’s exact test.

**Results:**

The proportion of respondents in 2020 who agreed to reduce serum uric acid levels below 6 mg/dL in gout patients with tophi significantly increased compared to 2018. The agreement in 2020 with the long-term use of colchicine to reduce the recurrence of gout flares when initiating urate-lowering agents (ULAs) significantly increased compared to 2018. There were no significant differences between 2018 and 2020 in clinicians’ attitudes toward support for the third JGMHG in daily practice, support for the use of recommendations toward each CQ, or support for finding themes for research or important clinical issues.

**Conclusions:**

The third JGMHG plays an important role in popularizing recommendations for serum uric acid levels in patients with tophi and the long-term use of colchicine for prophylaxis of gout flares when initiating ULA, although it did not influence the distribution of responses regarding clinicians’ attitudes toward its support.

## Introduction

Clinical practice guidelines (CPGs) influence decision-making in daily practice ^(1), (2)^. Although some studies have shown that CPGs improve the quality and consistency of healthcare ^(3), (4), (5), (6)^, there is concern that CPGs do not always yield predicted improvements. Common criticisms include CPGs that oversimplify practice and encourage litigation ^(7)^. These attitudinal issues can limit implementation. Therefore, it is necessary to follow up on the usage of CPGs and evaluate how CPGs could influence decision-making in daily practices. Indeed, to develop high-quality CPG, the Appraisal of Guidelines for Research and Evaluation II (AGREE II) recommends monitoring to clarify how CPG can be leveraged not only in clinical settings but also in society ^(8)^. Several reports have been published on the results of CPG monitoring. In rheumatology, CPGs were generally well accepted, although the proportion of agreement decreased after the introduction of new guidelines ^(9)^. The two cross-sectional questionnaire surveys suggested concerns regarding the use of CPG in malpractice litigation. Therefore, CPG monitoring may be required.

The number of patients with gout and hyperuricemia in Japan is growing annually, reaching approximately 1.3 million and 10 million, respectively, by 2022. In addition to genetic background, the population increase could be attributed to a Westernized lifestyle, indicating that hyperuricemia and gout are common lifestyle-related diseases. The Japanese version of the Guideline for Management of Hyperuricemia and Gout (JGMHG) was established in 2002 for the first time in the world ^(10)^. Epidemiological and clinical data have accumulated since then, and the second edition of the JGMHG was published in 2010, which emphasized the importance of uric acid as a risk and/or marker of complications, such as cerebro-cardio-renal-artery diseases ^(11)^. Several mechanisms have recently been proposed to explain the relationship between uric acid and/or monosodium urate monohydrate (MSU) crystals and cerebro-cardio-renal artery disease ^(12)^, and it is expected that intervention with urate-lowering agents (ULAs) may prevent organ dysfunction ^(13)^. To address the issue of whether ULAs could prevent organ dysfunction in asymptomatic hyperuricemia patients with kidney disease, hypertension, or heart failure, clinical questions (CQs) regarding the effects of ULAs on the prognosis of these patients have been summarized, and recommendations were established in the third edition of the JGMHG in 2018 ^(14)^.

The attitudinal barriers of practitioners toward CPGs include 1) the anti-intellectual to standardize practice around the average, 2) preventing discretion in individual cases, 3) cost-cutting, 4) limiting innovation, 5) clinical freedom, and 6) encouraging litigation ^(7)^. Thus, monitoring CPG users is required. Methods of monitoring CPGs have progressed since their development. This method is based on the Grading of Recommendations, Assessment, Development, and Evaluations approach, and the results of monitoring should be utilized to generate future CPGs ^(15), (16)^.

In the present report, we surveyed the practitioners’ attitudes toward the recommendations for CQs of the Japanese Guideline for Management of Hyperuricemia and Gout, third edition ^(14)^ in both 2018 and 2020 and evaluated their changes between 2018 and 2022 by means of the repeated questionnaire surveys.

## Materials and Methods

### Sample subjects

The survey participants were registered members of the Japanese Society of Gout, Uric, and Nucleic Acid (JSGUN). They included specialists in the treatment of gout and hyperuricemia, such as rheumatologists, orthopedic surgeons, nephrologists, cardiologists, diabetologists, endocrinologists, and general internists. The survey was sent via e-mail to eligible members, and replies were received; duplicate responses were excluded; 74 out of 463 (16.0% response rate) were collected in 2018, and 61 out of 436 (14.0% response rate) were collected in 2020, respectively (Table 1). There were no omissions in the questionnaires, and all were included in the analysis. This study was approved by the National Hospital Organization Yonago Medical Center Ethics Committee (Number 0609-02). Informed consent was obtained by an opt-out method. Those who rejected were excluded.

**Table 1.** Demographics of Eligible Members and Members Who Submitted Questionnaires for 2018 and 2020.

		Members who responded in 2018	Eligible members in 2018	Members who responded in 2020	Eligible members in 2020
		(n=74)	(n=463)	(n=61)	(n=439)
		n (%)	n (%)	n (%)	n (%)
Age	~39	9 (12.2%)	51 (11.0%)	3 (4.9%)	51 (11.7%)
	40~49	10 (13.5%)	73 (15.8%)	10 (16.4%)	69 (15.8%)
	50~59	23 (31.1%)	127 (27.4%)	22 (36.1%)	134 (30.7%)
	60~69	25 (33.8%)	99 (21.4%)	21 (34.4%)	115 (26.4%)
	70~	7 (9.5%)	50 (10.8%)	5 (8.2%)	59 (13.5%)
	Unknown	0 (0.0%)	63 (13.6%)	0 (0.0%)	8 (1.8%)
Sex	Women	4 (5.4%)	69 (14.9%)	3 (5.0%)	66 (15.1%)
	Men	70 (94.6%)	394 (85.1%)	58 (95%)	370 (84.9%)
Specialty	Internal Medicine	4 (5.4%)	191 (41.3%)	7 (11.5%)	207 (47.5%)
	Rheumatology	13 (17.6%)	45 (9.7%)	14 (23.0%)	25 (5.7%)
	Nephrology	17 (23.0%)	49 (10.6%)	11 (18.0%)	47 (10.8%)
	Cardiology	16 (21.6%)	35 (7.6%)	14 (23.0%)	23 (5.3%)
	Orthopedic surgery	6 (8.1%)	33 (7.1%)	2 (3.3%)	28 (6.4%)
	Diabetes, Metabolism and Endocrinology	10 (13.5%)	32 (6.9%)	7 (11.5%)	34 (7.8%)
	Hematology and Oncology	3 (4.1%)	13 (2.8%)	3 (5.0%)	10 (2.3%)
	Others	5 (6.8%)	65 (14.0%)	3 (5.0%)	62 (14.2%)
Affiliation	University	35 (47.3%)	198 (42.8%)	24 (39.3%)	156 (35.8%)
	Hospital	23 (31.1%)	128 (27.6%)	24 (39.3%)	138 (31.7%)
	Clinic	15 (20.3%)	130 (28.1%)	13 (21.3%)	138 (31.7%)
	Others	1 (1.4%)	7 (1.5%)	0 (0%)	4 (0.9%)

The demographic composition of age, sex, and affiliation among eligible and responding members was almost identical. However, there were fewer responses from members specializing in internal medicine. The majority of JSGUN members were male, with the largest age group being 50-59 years. Most members were affiliated with universities, followed by hospitals. JSGUN: Japanese Society of Gout, Uric, and Nucleic Acid.

### Survey Instruments

Supplemental Table 1 shows seven CQs and their recommendation in the third edition of the JGMHG ^(14)^. The first survey participants (n = 74) received questionnaires via e-mail between October and November 2018, just before the publication of the CPG. The second survey was sent via e-mail to physicians (n = 61) between October and November 2020. The questions (items #1 to 10) were the same between the 2018 and 2020 surveys. Both surveyors inquired about their recommendations for each CQ (items #1 to 7) and their experience using the CPG (items #8 to 10). Questions (items #11 to 17) were unique to the 2020 survey and included comprehensiveness of algorithms, the influence of the third edition JGMHG on therapeutic principles, usefulness in the clinical setting, new challenging issues, impact on confidence in the treatment, usefulness in decision-making, restriction of physician’s discretion, influence on the education of medical students and medical interns, and usefulness of communication tools with medical staff. Table 2 and 3 present details on the 17 questionnaires. The 5-point scale for answers ranging from “Strongly agree” to “Strongly disagree” was used.

**Table 2.** Details on the Ten Common Questionaries Regarding the Use and Attitudes Toward CPGs between 2018 and 2020 and Proportions of Agreement and/or Disagreement among Responders in 2020.

Items No	Questionnaire of the Use Toward CPG	Agree to ①	Agree to ②	Agree to ③	Agree to ④	Agree to ⑤
		n (%)	n (%)	n (%)	n (%)	n (%)
1	Which agent among NSAIDs, glucocorticoids, and colchicine is most favorable for gout flares? ①NSAID, ②glucocorticoid, ③colchicine, ④three agents are equal, ⑤I do not know.	31 (50.8)	3 (4.9)	8 (13.1)	14 (23.0)	5 (8.2)
**Items No**	**Questionnaire of the Use of and Attitudes Toward CPG**	**Strongly agree and Agree groups**		**Disagree, Strongly disagree, and Other groups**		
		**Number (%)**		**Number (%)**		
2	Do you use ULA to preserve renal function in hyperuricemic patients with kidney disease? ①strongly agree, ②agree, ③disagree, ④strongly disagree, ⑤ I do not know.	53 (86.9)		8 (13.1)		
3	Do you use ULA to improve patient prognosis and reduce the cardiovascular event risk in hyperuricemic patients with hypertension? ①strongly agree, ②agree, ③disagree, ④strongly disagree, ⑤ I do not know.	27 (44.3)		34 (55.7)		
4	Is your target serum urate level for treating gout patients with tophi less than 6 mg/dl? ①strongly agree, ②agree, ③disagree, ④strongly disagree, ⑤ I do not know.	57 (93.4)		4 (6.6)		
5	Do you use ULA to improve the patient’s prognosis in hyperuricemic patients with heart failure? ①strongly agree, ②agree, ③disagree, ④strongly disagree, ⑤ I do not know.	23 (37.7)		38 (62.3)		
6	Do you use colchicine coverage to prevent recurrence of gout flares in gout patients when initiating ULA? ①strongly agree, ②agree, ③disagree, ④strongly disagree, ⑤ I do not know.	33 (54.1)		28 (45.9)		
7	Do you provide dietary education to asymptomatic hyperuricemic patients? ①strongly agree, ②agree, ③disagree, ④strongly disagree, ⑤ I do not know.	42 (68.9)		19 (31.1)		
8	Do you use the guidelines for the management and treatment of hyperuricemia and gout in your daily practice? ①strongly agree, ②agree, ③disagree, ④strongly disagree, ⑤ I do not know.	53 (86.9)		8 (13.1)		
9	Do you find the recommendations for CQ 1 to 7 useful in your daily practice? ①strongly agree, ②agree, ③disagree, ④strongly disagree, ⑤ I do not know.	55 (90.2)		6 (9.8)		
10	Did this guideline help you identify a theme for your clinical research or an important clinical issue for future CQs? ①strongly agree, ②agree, ③disagree, ④strongly disagree, ⑤ I do not know.	49 (80.3)		12 (19.7)		

Item 1 (the questionaries) regarding CQ1 asked which agent among NSAIDs, glucocorticoids, and colchicine was most favorable for gout flares. CQ2 inquired about how often ULA would be used to preserve renal function in hyperuricemic patients with kidney disease. The questionaries of CQ3 inquired about how often ULA would be prescribed to reduce the risk of cardiovascular events in patients with hyperuricemia and hypertension. The questions in CQ4 inquired whether the target SUA level should be <6 mg/dL for gout patients with tophus. The questions in CQ 5 asked how often ULA would be used to improve the prognosis of hyperuricemic patients with heart failure. The CQ6 questionnaire inquired whether colchicine coverage would be applied to prevent the recurrence of gout flares in patients with gout who underwent ULA. Question 7 regarding CQ7 inquired whether diet education could be applied to asymptomatic patients with hyperuricemia. Question 8 asked whether the current guidelines for the management of hyperuricemia and gout were utilized in the clinical setting. Question 9 asked whether the recommendation of CQ 1 to 7 was useful in daily practice. Question 10 asked whether the current guidelines would include either future issues for clinical research or important clinical issues for future CQs. In addition, the proportions of agreement and/or disagreement among 2020 respondents from CQ2 to CQ10, respectively. CPG: Clinical practice guideline; CQ: clinical question; NSAIDS: non-steroidal anti-inflammatory drugs; ULA: urate-lowering agent.

**Table 3.** Details on the Seven New Questionaries in 2020 regarding the Use and Attitudes toward CPGs and Proportions Of Agreement and/or Disagreement among Responders in 2020.

Items No	Questionnaire on the Use of and Attitudes Toward CPG	Strongly agree and Agree groups (%)	Disagree, Strongly disagree, and Other groups (%)
11	Are the algorithms of this guideline comprehensive? ①strongly agree, ②agree, ③disagree, ④strongly disagree, ⑤ I do not know.	56 (91.8)	5 (8.2)
12	Did you change your therapeutic approach after using this guideline? ①strongly agree, ②agree, ③disagree, ④strongly disagree, ⑤ I do not know.	41(67.2)	20 (32.8)
13	Did you gain more confidence in the treatment of hyperuricemia and gout after using this guideline? ①strongly agree, ②agree, ③disagree, ④strongly disagree, ⑤ I do not know.	45 (73.8)	16 (26.2)
14	Do you use this guideline to inform your decision-making with patients? ①strongly agree, ②agree, ③disagree, ④strongly disagree, ⑤ I do not know.	43 (70.5)	18 (29.5)
15	Do you think this guideline may restrict the discretion of the physician? ①strongly agree, ②agree, ③disagree, ④strongly disagree, ⑤ I do not know.	15 (24.6)	46 (75.4)
16	Do you use this guideline for educating medical students and medical interns? ①strongly agree, ②agree, ③disagree, ④strongly disagree, ⑤ I do not know.	31 (50.8)	30 (49.2)
17	Do you use this guideline to communicate with medical staff? ①strongly agree, ②agree, ③disagree, ④strongly disagree, ⑤ I do not know.	28 (45.9)	33 (54.1)

Question 11 inquired how comprehensive the algorithms of the current guidelines would be. Question 12 asked whether the current guidelines may have a deep impact on your treatment decision-making. Question 13 asked whether the current guidelines facilitated confidence in the treatment of hyperuricemia and gout. Question 14 asked how often the current guidelines were used for decision-making with patients. Question 15 asked whether the current guidelines restrict the discretion of the physician. Question 16 asked whether the current guidelines could be used to educate medical students and medical interns. Question 17 inquired whether the current guidelines were used to communicate with the medical staff. Question 18 inquired about requests for opinions on the current CPG. CPG: Clinical practice guideline.

### Statistical Analysis

Statistical analysis was performed using EZR software (Easy R, version 1.68). Categorical variable distributions were compared using the chi-square test or Fisher’s exact test.

A p-value of <0.05 was considered to indicate a significant difference.

## Results

### Comparisons of the Proportion of Agreement on a Recommendation to Common Questionaries between 2018 and 2020

**Figure 1.**
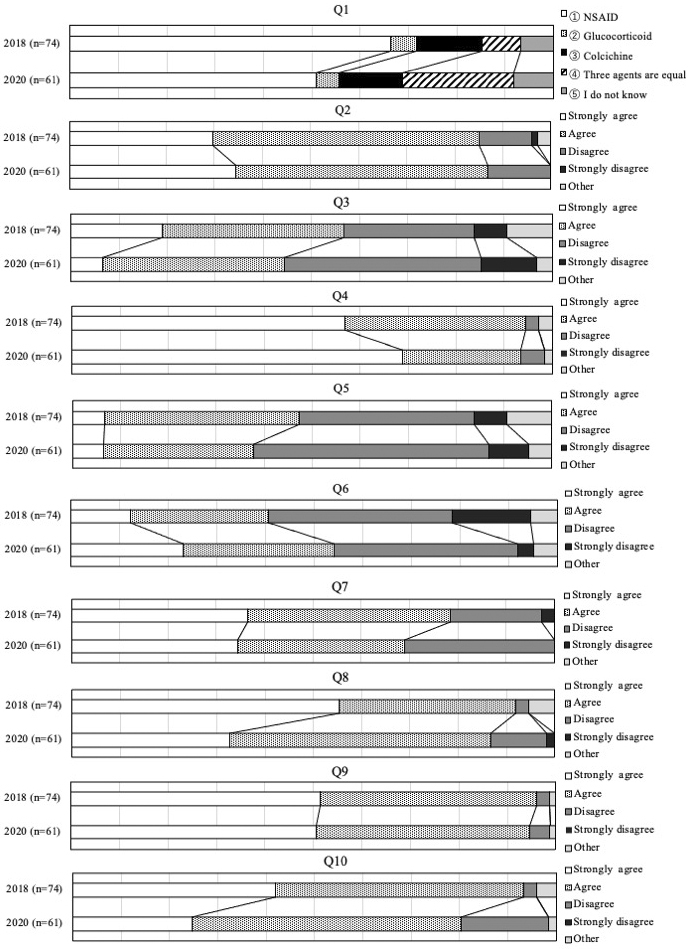
Comparison of the distribution of the proportions of both agreement and/or disagreement of the respondents toward each questionnaire survey from Q1 to 10 between 2018 and 2020. Q: question.

Table 2 shows the common questions from Questions 1 to 10 (Q1 to 10) in 2018 and 2020 and the proportions of agreement and/or disagreement among responders in 2020. While the proportion of agreement of respondents toward the use of anti-inflammatory agents in Q1 was highest at 50.8%, the proportion of agreement on recommendations in Q2 to 7 varied from 37.7% to 93.4%. Figure 1 compares the distribution of agreement and/or disagreement for each questionnaire from Q1 to 10 between 2018 and 2020. The chi-square test in Q4 indicated that there was a significant difference in the distribution of strong agreement regarding the target to treat serum uric acid (SUA) levels below 6 mg/dL in gout patients with tophi. The proportion of strong agreement increased from 56.8% in 2018 to 68.9% in 2020. Fisher’s exact test in Q6 indicated that there was no significant difference in the distribution of responses regarding prophylaxis with colchicine to reduce gout flairs after initiating ULA, although the proportions of both strong agreement and agreement significantly increased from 40.5% in 2018 to 54.1% in 2020. Fisher’s exact test in Q1 indicated that there was no difference in the distribution of responses to the usage of any anti-inflammatory agent. However, agreement on the use of colchicine, or any agent including colchicine, significantly increased from 21.6% in 2018 to 36.1% in 2020. Fisher’s exact test in Q2, 3, and 5 indicated that there was no difference between 2018 and 2020 in the distribution of responses to the use of ULA to prevent organ dysfunction in patients with asymptomatic hyperuricemia associated with kidney disease, hypertension, or heart failure. Fisher’s exact test for Q7 indicated no difference between 2018 and 2020 in the distribution of responses to the application of dietary education.

Agreement on clinicians’ attitudes toward supporting the third edition of the JGMHG in daily practice, supporting the use of recommendations for each CQ, and supporting research theme identification or the identification of important clinical issues (Q8 to 10) ranged from 80.3% to 90.2% in 2020. Fisher’s exact test for Q8, 9, and 10 indicated no significant differences in the distribution of clinician response attitudes between 2018 and 2020.

### General Attitude Toward the Third Edition JGMHG in Responders to New Questionaries in 2020

Table 3 shows the proportion of agreement with the new questionaries in 2020 (Q11 to 17) regarding general attitudes toward the third edition of the JGMHG. The proportion of both strong agreement and agreement among respondents to the questionaries on the comprehensiveness of the algorithm, effects on therapeutic principles, effects on treatment confidence, effects on decision-making, restrictions on physician discretion, usefulness for education, and effects on communication with medical staff were 91.8%, 67.2%, 73.8%, 70.5%, 24.6%, 50.8%, and 45.9%, respectively.

## Discussion

In the present study, we found that: 1) the proportion of strong agreement in 2020 regarding the treatment target of an SUA level below 6 mg/dL in gout patients with tophus increased compared to 2018; 2) the proportion of both strong agreement and agreement in 2020 regarding the use of colchicine prophylaxis to reduce the recurrence of gout flares when initiating ULA increased compared to 2018; 3) the proportion of agreement in 2020 regarding the use of colchicine increased compared to 2018; 4) there was no significant difference in the proportion of agreement on the selection of anti-inflammatory agents for gout flares, use of ULA to prevent patients with kidney disease, hypertension and heart failure from organ damage or application of dietary education; 5) there were no significant differences in the distribution of responses to clinicians’ attitudes toward support for the third edition of the JGMHG in daily practice, support for recommendations regarding each CQ, or support for identifying themes for research or important clinical issues between 2018 and 2020.

Febuxostat significantly reduced the size of tophi in gout patients with SUA levels <6 mg/dL compared to allopurinol ^(17)^. Pegloticase reduced SUA to less than 6 mg/dL and reduced the size of tophi by 40% compared to placebo ^(18)^, whereas lesinurad and allopurinol, which also reduced SUA to less than 6 mg/dL, resulted in complete remission of tophi ^(19)^. Taken together, the third edition of the JGMHG recommends reducing SUA to <6 mg/dL in the treatment of patients with tophi ^(10)^. In the present study, agreement in 2020 on the target SUA level below 6 mg/dL in gout patients with tophi increased compared to 2018, suggesting that the third edition of the JGMHG influenced the practice of tophus treatment. There is a discrepancy in the target treatment of SUA in patients with gout and tophus between Japan and Western countries. While the third edition of the JGMHG recommends lowering SUA levels to <6 mg/dL, both the EULA and US CPGs recommend a target of <5 mg/dL ^(20)^. Further research is necessary to reevaluate the target SUA values.

Long-term administration of colchicine from 7 to 9 months, compared to short-term administration from 3 to 6 months, significantly reduced recurrence (from 54% to 27.4%) ^(21)^. However, long-term administration of colchicine significantly increased the incidence of liver dysfunction compared to short-term administration. Post-hoc analysis using RCTs such as FACT ^(17), (22)^, APEX ^(17), (22)^, and CONFIRM ^(23)^, conducted by members of the third edition of the JGMHG, found that long-term administration of colchicine for 6 months reduced recurrence of gout flares after initiation of ULA (4%) compared with short-term administration of colchicine for 2 to 3 months (33.5%). However, adverse effects, such as liver dysfunction, were significantly higher in patients treated with colchicine for the long term (7.7%) than in those treated for the short term (2.5%) ^(10)^. The proportion of agreements on the long-term use of colchicine to reduce the recurrence of gout flares increased in 2020 compared to that in 2018, suggesting that the third edition of the JGMHG influenced the practice of gout flares prophylaxis. Before the publication of the third edition of the JGMHG, because of the adverse effects of colchicine, such as diarrhea and agranulocytes, physicians in Japan hesitated to use colchicine for the prophylaxis of gout flares. However, the present guidelines show the beneficial effects of long-term administration of colchicine, which might be attributable to the increase in favorable responses to the use of colchicine in this survey.

Interestingly, both strong agreement and agreement were higher in Q2 regarding the usefulness of ULAs for preventing renal dysfunction in hyperuricemic patients with chronic kidney disease (CKD) than in Q3 and Q5 regarding the usefulness of ULAs in reducing mortality in hyperuricemic patients with hypertension and heart failure. Although various clinical trials ^(13)^ have verified the effects of ULAs on the prognosis and kidney function of patients with kidney disease, few studies have verified the effects of ULAs on the prognosis of patients with either hypertension or heart failure. Both strong agreement and agreement in Q7 regarding the usefulness of dietary education tended to be higher in 2020 than in 2018. CQ7 focused on ingredients; however, it did not include the usefulness of dietary education. Further studies are required to address this issue.

In terms of new questions in 2020, the members of JSGUN had comparable or greater support for the third edition of the JGMHG than the average level reported in a systematic review described elsewhere in a survey conducted between 1990 and 2000 ^(24)^. Although intended to aid practice, CPGs have also been shown to cause mixed reactions among practicing physicians. Common criticisms include the statement that CPGs may oversimplify their practices ^(25)^. Some physicians are concerned that CPG may be used to bring malpractice actions against physicians, while others feel that it threatens the autonomy of physicians and the patient-physician relationship ^(7)^. The proportions of agreement on opinions that guidelines were useful sources of advice, good educational tools, and intended to improve quality were 75%, 71%, and 70%, respectively ^(24)^. A sizable minority believed that the guidelines reduce physician autonomy. In the present study, although the wording of the questionnaires was slightly different from others, the proportion of agreement regarding the opinions that the guidelines are useful sources of advice, good educational tools, and intended to improve quality were 88.3%-91.7%, 51.7%, and 66.7%-73.3%, respectively. These findings suggest that members of the JSGUN have comparable or greater support for CPGs than the average level reported in a systematic review ^(24)^ of surveys conducted between 1990 and 2000. Furthermore, negative views represented by the proportion of respondents who felt that CPGs restricted physician autonomy at 25% were comparable or smaller than the average in Farquhar’s systematic review (22%-35%) ^(24)^. The positive opinions in the present study may be attributed to the underlying trust in these CPGs. However, further studies are required to clarify these differences.

Limitations: One of the limitations of this survey was the low response rate to the questionnaires, with less than 20% participation for each. This likely meant that only members with a particular interest in the guidelines responded, introducing potential bias. Consequently, there is concern that the views of guideline users as a whole may not be adequately reflected. There were omissions in the membership registration of JSGUN members, which meant that the ages and other details of eligible members could not be fully collected. In addition, the proportion of agreement regarding the guidelines as good educational tools was 52%, slightly lower than the 71% reported by Farquhar et al. ^(24)^. We are currently working on the 4th edition of the JGMHG and intend to use these results to make the guidelines more accessible in clinical and educational settings.

In conclusion, the third edition of the JGMHG plays an important role in promoting recommendations for targeting SUA levels in patients with tophi and the long-term use of colchicine for gout flares prophylaxis when initiating ULA. However, it did not significantly influence the distribution of responses regarding clinicians’ attitudes.

## Article Information

### Conflicts of Interest

Naoyoshi Otani, Toshihiro Hamada, Masanari Kuwabara, Satoshi Miyazaki, Yasuto Sato, and Haruaki Ninomiya declare no conflict of interest. Takeo Nakayama reports grants or contracts: I&H Co., Ltd.; Cocokarafine Group Co., Ltd.; Konica Minolta, Inc.; NTT DATA; consulting fees: Ohtsuka Pharmaceutical Co.; Takeda Pharmaceutical Co.; Johnson & Johnson K.K.; AstraZeneca plc; Nippon Zoki Pharmaceutical Co., Ltd.; payment or honoraria for lectures, presentations, speakers bureaus, manuscript writing or educational events: Pfizer Japan Inc.; MSD K.K.; Chugai Pharmaceutical Co.; Takeda Pharmaceutical Co.; Janssen Pharmaceutical K.K.; Boehringer Ingelheim International GmbH.; Eli Lilly Japan K.K.; Maruho Co., Ltd.; Mitsubishi Tanabe Pharma Co.; Novartis Pharma K.K.; Allergan Japan K.K.; Novo Nordisk Pharma Ltd.; TOA EIYO Ltd.; AbbVie Inc.; ONO PHARMACEUTICAL CO., LTD.; GSK plc; Alexion Pharmaceuticals, Inc.; Cannon Medical Systems Co.; Kowa Company, Limited; Araya; Merck Co.; Amicus Therapeutics, Inc.; stock options: BonBon inc.; donation: CancerScan; JMDC Inc. TK received lecture fees from Mochida Pharmaceutical, Fujiyakuhin, Sanawa kagaku, Nippon Chemiphar, and Boehringer Ingelheim. IH received research grants from Mochida Pharmaceutical, Meiji, and Fujiyakuhin and lecture fees from Mochida Pharmaceutical, Fujiyakuhin.

### Acknowledgement

We would like to thank the staff of the Japanese Society of Gout, Uric, and Nucleic Acid for their assistance in collecting the data for preparing this manuscript.

### Author Contributions

Concept: Naoyoshi Otani, Tsuneo Konta, and Ichiro Hisatome. Data collection: Naoyoshi Otani, Toshihiro Hamada, Masanari Kuwabara, and Satoshi Miyazaki, Discussion: Yasuto Sato, Takeo Nakayama, and Haruaki Ninomiya. All authors have read and agreed to the published version of the manuscript.

### Approval by Institutional Review Board (IRB)

No. 0609-02 and NHO Yonago Medical Center, Ethics Committee.

## Supplement

Supplemental Table 1 The seven CQs and their recommendations.
