# Supplementary material for: Changes in Practitioners’ Attitudes toward the Recommendations for Clinical Questions of the Japanese Guideline for Management of Hyperuricemia and Gout, Third Edition between 2018 and 2020 in the Questionnaire’s Surveys: Involving Their Attitude toward the New Questionnaires in 2020
Source: JMA J. 2025 Mar 21;8(2):470–8. doi: 10.31662/jmaj.2024-0399 (PMC12095857; doi:10.31662/jmaj.2024-0399)
Supplement: Supplemental Table 1 — The seven CQs and their recommendations. [file 2433-3298-8-2-0470-s001.pdf]

Supplemental Table 1: List of Clinical Questions (CQs) and Recommendations

	Clinical Questions (CQs)		Recommendations
CQ1	Can NSAIDs, glucocorticoids, and colchicine be recommended for patients with acute gouty arthritis (gout attacks) over no pharmacological treatment?	Recommendation 1	NSAIDs, glucocorticoids, and colchicine (low dose) are conditionally recommended for patients with acute gouty arthritis (gout attacks) over no pharmacological treatment.
CQ2	Can urate-lowering agents be recommended in patients with hyperuricemia and kidney injury over non-pharmacological treatment?	Recommendation 2	The use of urate-lowering agents to retard the decline in kidney function is conditionally recommended in patients with hyperuricemia and kidney injury.
CQ3	Can urate-lowering agents be recommended for hypertensive patients with hyperuricemia over non-pharmacological treatment?	Recommendation 3	The use of urate-lowering agents to improve life prognosis and reduce the risk of cardiovascular disease cannot be actively recommended for hypertensive patients with hyperuricemia.
CQ4	Can one recommend maintaining the serum urate concentration at ≤6.0 mg/dL with pharmacotherapy for patients with tophi?	Recommendation 4	It can be recommended to maintain the serum urate concentration at ≤6.0 mg/dL with pharmacotherapy for patients with tophi.
CQ5	Can urate-lowering agents be recommended for patients with heart failure and hyperuricemia over non-pharmacological treatment?	Recommendation 5	The use of urate-lowering agents to improve life prognosis and reduce the risk of cardiovascular disease cannot be actively recommended for patients with heart failure and hyperuricemia.
CQ6	Can long-term colchicine use be recommended over short-term use to prevent gout attacks in gout patients after initiating urate-lowering therapy?	Recommendation 6	Long-term colchicine use to prevent gout attacks can be conditionally recommended for gout patients when starting urate-lowering therapy.
CQ7	Can dietary advice be recommended over not providing dietary advice to patients with asymptomatic hyperuricemia?	Recommendation 7	Dietary advice is recommended over not providing dietary advice to patients with asymptomatic hyperuricemia
